# Epidemiological study of patients with facial trauma treated at the Antônio Targino Hospital - Campina Grande/Paraíba

**DOI:** 10.1016/S1808-8694(15)30509-7

**Published:** 2015-10-18

**Authors:** Josuel Raimundo Cavalcante, Karis Barbosa Guimarães, Belmiro Cavalcanti do Egito Vasconcelos, Ricardo José de Hollanda Vasconcellos

**Affiliations:** 1 MSc, PhD student - FOP/UPE, Adjunct Professor - UEPB; 2 MSc. PhD student - FOP/UPE, Assistant Professor - UFPB; 3 PhD, Graduate Studies Coordinator - UPE; 4 PhD, Adjunct Professor - FOP/UPE. PhD Program in Dentistry - Maxillo-Facial Surgery and Trauma - Universidade of Pernambuco

**Keywords:** surgery, health services epidemiology, oral

## Abstract

Information contained in the present study can better explain the type of care performed in this service, which is paramount in order to plan, organize and improve medical care here.

**Aim:**

the goal of the present study was to carry out a retrospective epidemiological study concerning facial trauma from August 2006 to August 2007.

**Materials and Methods:**

211 charts were studied in order to collect patient data regarding the number of patients seen, gender, age, year and their origin, surgical approaches and cases.

**Results:**

Among the surgical cases, facial fractures were the most prevalent (73.9%). Male patients prevailed (81.5%), in the ages between 11 and 40 years from the arid Paraíba mesoregion. The most frequent causes we motorcycle accidents, making up 64.5% of the sample, and the zygomatic-orbital complex was the most affected site.

**Conclusion:**

The most affected patients were males, and those from the Paraíba arid mesoregion were the ones who benefited the most from the service. Among the most frequent etiological factors we list: motorcycle accidents, physical fights and falls from one's own height.

## INTRODUCTION

Healthcare Services is a general term given to sites designed for health promotion, protection or recovery, for outpatients or inpatients, regardless of the complexity level. The morbidity profile of the population should determine, by and large, the type, quantity and distribution of services made available to the collectivity, to maintain or improve health status^1^.

Epidemiology can and may be used for administration of health services. It contributes to establish diagnosis of a community, as well as presence, nature and distribution of health and disease^2^.

The data produced by operation of services give subsidies for decision-making processes since they enable - if properly dealt with - a collective and evolutionary view of problems, indicating the paths to improve care and decrease costs^2^. Moreover, quantitative information about supply of these services are valuable for structural planning, to better understand the care patterns practiced and to debate about future provision of new services, in addition to training needed to exercise a specialty^3^, ^4^.

The facial lesions, including fractures, play a significant role in care of polytrauma patients in hospital emergencies. Some studies conducted with the objective of outlining an epidemiological profile of facial traumas all over the world correlate to social, urban and rural changes as modifying agents of interpersonal relationships, generating physical violence actions, of personal and group character, represented by physical aggressions, violence in transit, domestic violence and violence against women^5^.

## OBJECTIVE

Due to diverse data in the literature and peculiarities of each region studied, the objective of the present investigation was to carry out a retrospective epidemiological study of patients seen at a hospital trauma service, to make a survey of services delivered to the community, as well as characterization of patient profile, in the period between August 2006 and August 2007.

## MATERIAL AND METHODS

It is an epidemiological, descriptive and retrospective study. Data of patients who went to the Trauma Service of Hospital Antônio Targino, Campina Grande, Paraiba - Brazil, were colleted in the period between August 2006 and August 2007. The patients' data were obtained from hospital files, and all patients seen at outpatient clinic and hospital (inpatients and/or operated on) were selected.

This study was performed after approval by the Ethics Committee of the Universidade Estadual da Paraíba, under number 0105.011331000-08.

After selecting the sample, a worksheet was prepared to collect data, which were organized in a database using the software Epi Info®, version 3.4.1, for descriptive analysis, and the final sample comprised 211 patients. After collecting data, the results were submitted to descriptive analysis.

## RESULTS

Out of 211 patients analyzed in this study, 172 (81.5%) were male and 39 (18.5%) female. The mean age was 36.84 years. The age group 21-30 years was the most prevalent, followed by 11-20 years, and both groups represented 58.3% of patients. The distribution of patients per age group and gender is described on Table 1.

**Table 1:** Distribution of sample per age group and gender of patients.

Gender	0-10	11-20	21-30	31-40	41-50	51-60	61-70	71-80	Not Informed	TOTAL
Male %	4	42	60	26	22	8	6	4	0	172
	2,3	24,4	34,9	15,1	12,8	4,7	3,5	2,3	0,0	81,5
Female %	1	9	12	7	6	2	1	0	1	39
	2,6	23,1	30,8	17,9	15,4	5,1	2,6	0,0	2,6	18,5
TOTAL %	5	51	72	33	28	10	7	4	1	211
	2,4	24,2	34,1	15,6	13,3	4,7	3,3	1,9	0,5	100,0

Source: Data of the study. 2007.

A tendency to maintain the number of visits throughout time was observed in the group of patients studied, because in 2006 only four months were assessed and, in 2007, eight months (Table 2).

**Table 2:** Distribution and frequency of the population per year and gender of patients.

Year	Male	Female	TOTAL
2007 (%)	117 (80,7)	28 (19,3)	145 (68,7)
2006 (%)	55 (83,3)	11 (16,7)	66 (31,3)
TOTAL (%)	172 (81,5)	39 (18,5)	211 (100,0)

Source: Data of the study. 2007.

When analyzing the origin of patients, divided according to the Brazilian Institute of Geography and Statistics (IBGE) into geographic microregions of the State of Paraiba (zona da mata, agreste, borborema and sertão), there was a predominance of patients living in the agreste region, corresponding to 56.9% of patients, as shown in Table 3. Out of 120 (56.9%) patients coming from the agreste region, only 41 (34.16%) lived in the city of Campina Grande.

**Table 3:** Distribution and frequency of the population per region of residence.

Mesoregion	Frequency	Percentage
Zona da Mata	2	0,9%
Agreste	120	56,9%
Borborema	30	14,2%
Sertão	46	21,8%
Other states	11	5,2%
Not informed	2	0,9%
TOTAL	211	100,0%

Source: Data of the study. 2007.

As to etiological factors, motorbike accidents prevailed, representing 64.5% of sample, followed by physical aggressions and falls, summing up 11.4% and 10.4%, respectively. The etiological factors are displayed in Table 4.

**Table 4:** Distribution of the sample per etiology and gender of patients.

Etiology	Male	Female	TOTAL
Motorbike accident (%)	113 (83,1)	23 (16,9)	136 (64,5)
Road accident (%)	8 (80,0)	2 (20,0)	10 (4,7)
Weapon injury (%)	3 (75,0)	1 (25,0)	4 (1,9)
Physical aggression (%)	19 (79,2)	5 (20,8)	24 (11,4)
Accidents with animals (%)	2 (100,0)	0 (0,0)	2 (0,9)
Falls (%)	15 (68,2)	7 (31,8)	22 (10,4)
Bicycle accidents (%)	8 (88,9)	1 (11,1)	9 (4,3)
Sports accidents (%)	1 (100,0)	0 (0,0)	1 (0,5)
Not informed (%)	3 (100,0)	0 (0,0)	3 (1,4)
TOTAL (%)	172 (81,5)	39 (18,5)	211 (100,0)

Source: Data of the study. 2007.

Regarding the type of procedure performed, surgeries for facial fractures were the most frequent, followed by suture of small soft tissue lesions (Table 5). Analyzing surgeries to reduce facial fractures, which accounted to 73.9% of procedures performed in the service, the age groups 11-20 and 21-30 years predominated, totaling up 55.8% of patients. Considering the type of lesion, orbitozygomatic complex and mandibular fractures prevailed accounting for 52.6% of patients. Concerning the small lesions, partial lip reconstructions and gingivolabial sulcus stood out (Table 6).

**Table 5:** Distribution and frequency of the population per type of lesion and gender.

Type of lesion	Frequency	Percentage	Male	Female
Dentoalveolar trauma	4	1,89%	4 (1,89%)	0 (0,0%)
Facial fractures	156	73,93%	125 (59,24%)	31 (14,69%)
Suture of wounds	51	24,17%	43 (20,37%)	8 (3,79%)
TOTAL	211	100,0%	172 (81,51%)	39 (18,48%)

**Table 6:** Distribution and frequency of the population per characteristics and anatomical sites of lesions.

Type of lesion	Frequency	Percentage
Dentoalveolar fracture	4	1,9%
Orbit fracture	9	4,3%
Orbitozygomatic complex fracture	56	26,5%
Mandibular fracture	55	26,1%
Maxillary fracture - Le Fort I	1	0,5%
Maxillary fracture - Le Fort II	2	0,9%
Maxillary fracture - Le Fort III	2	0,9%
Fracture of nasal bones	29	13,7%
Laceration/blunt injury - lip	20	9,5%
Laceration/blunt injury - gingivolabial sulcus	13	6,2%
Laceration/blunt injury - nose	4	1,9%
Laceration/blunt injury - palate	1	0,5%
Laceration/blunt injury - ear	1	0,5%
Laceration/blunt injury - eyelid	6	2,8%
Foreign body in mandible	2	0,9%
Wound with loss of substance	6	2,8%
TOTAL	211	100,0%

Source: Data of the study. 2007.

Assessing the procedures carried out for surgical reduction of facial fractures, the study showed that facial fracture surgeries (92.49%) and others (7.46%) were often submitted to intervention by open procedure (Table 7).

**Table 7:** Distribution and frequency of the population per surgical treatment, open surgery, types of lesions.

Surgical Treatment - Open surgery - Lesion	Frequency	Percentage
Orbit fracture	9	8,41%
Orbitozygomatic complex fracture	49	45,79%
Mandibular fracture	35	32,71%
Maxillary fracture - Le Fort I	1	0,93%
Maxillary fracture - Le Fort II	2	1,86%
Maxillary fracture - Le Fort III	2	1,86%
Fracture of nasal bones	1	0,93%
Foreign body in mandible	2	1,86%
Wound with loss of substance	6	5,60%
TOTAL	107	100,0%

Source: Data of the study. 2007.

## DISCUSSION

Epidemiological studies on facial traumas are well reported in the international literature^6^, ^7^. However most reports focus specifically on a certain type of care provided by a service to the community^8^. Few descriptions comprehensively explore the type of general care and the characteristics of patients submitted to maxillofacial interventions^3^, ^8^, ^9^.

As to gender, many reports in the international literature state females are more prevalent^10^. The rate of 18.5% (n= 39) of female patients found in this study contradicts the literature, and is in disagreement with the studies by Brennan et al.^3^, Ferraro-Bezerra^8^, Waldman^10^, Manski, Moeller and Hupp^11^; however, it agrees with the studies by Falcão, Leite Segundo and Silveira^5^ and Hill et al.^12^.

Specifically evaluating the surgical modalities and correlating with gender, there was a prevalence of males in all modalities. Associating types of surgery (facial fractures and others) with gender of the population studied, primarily males, the present study corroborates the findings of Ferraro-Bezerra^8^.

The literature is virtually unanimous showing that facial trauma patients are mostly young adults, aged 10-40 years, in several surgical modalities^8^, ^11^, ^13^. The mean age of 36.4 years observed in the present study corroborates the literature. This age group relatively low is justified by the fact that most surgical interventions are elective.

The findings of this investigation show that 56.9% (n=120) of patients seen at the service came from the agreste region, in Paraiba, in the mesoregion where the city of Campina Grande is located. This is due to the fact the hospital studied is considered a reference in this region for facial trauma.

Table 4 presents the distribution of sample per reason patients went to the hospital. Among the main causes, motorbike accidents contributed with 64.5%, followed by physical aggressions (11.4%) and falls (10.4%). Leite Segundo et al.^14^ demonstrated road accidents, followed by physical aggressions as the main etiological agents that triggered stomatognathic complex disorders, and these findings agree with ours.

Considering several types of treatment in facial trauma, fractures represent one of the major fields of study in epidemiology of healthcare services. There are many studies in the literature in which the demographic distribution of patients with trauma facial is analyzed by several criteria^15^. This field was represented by a relevant share of the population in the present study - approximately 75% of patients.

A high prevalence of male patients was observed (80.6%), and other studies on the topic corroborate this finding^7^, ^8^, ^16^. The main reason described in the literature for this male predominance is that men are more predisposed to risk factors, such as physical aggressions, road accidents, falls and accidents in sport activities^5^, ^16^, ^17^.

The more prevalent sites of fractures were the orbitozygomatic complex and mandibles, in 26.5% and 26.1% of patients, respectively. Nasal fractures ranked second. Although mandible fractures were more prevalent^7^, ^17^, the present study differs from reports due to the similar proportion of mandibular and orbitozygomatic complex fracture, but is in agreement with the studies by Falcão et al.^5^ and Ferraro-Bezerra^8^. The literature revised shows a major predominance of mandible fracture, and the investigations by Erol, Tanrikulu and Görgün^15^ and Motamedi^17^ reported a frequency of 72.9% and 72.8%, respectively. The mandible is the anatomical region with more interruptions and this fact is possibly explained for being the only mobile facial bone, being more vulnerable to receive strong impact and fracture.

Analyzing the types of surgery for facial fractures, open approach with reduction and posterior fixation of the bone stumps using titanium plates and screws was frequently applied and represented 66.04% of lesions. The introduction of new fixation systems, specifically the stable internal fixation, has provided stability needed to maintain the bone stumps in the original anatomical position, resulting from anatomical reduction^18^. Titanium plates and screws have been widely used for over two decades for stable internal fixation of facial fractures^19^. Therefore, the service analyzed complies with the principles of bone stump fixation through reduction and use of titanium plates and screws.

The outpatients visits for suture of small wounds and repair of soft tissues affected accounted for 24.17% in the population studied. Partial repair of lips and gingivolabial sulcus prevailed among these surgeries. It is noteworthy mentioning that outpatient care reduces hospital costs in such surgeries, since patients are discharged soon after the procedures.

The surgeries characterized as foreign body in the mandible and lesions with loss of substance in the maxillofacial region had a low prevalence during the period of study. They represented 7.46% (8) of total number of patients, and considering facial fractures as the most frequent cause, the hospital is confirmed as a reference for care of trauma patients. The study of Ferraro-Bezerra8 also demonstrated a lower rate (16.7%) for this treatment as compared to the total sample.

Based on these data, one can have an overview of care delivered to trauma. Hence, this study has an impact the management of services, contributing to better understand the type of care delivered.

## CONCLUSION

According to the methodology used and results obtained, the following conclusions may be drawn:

1. Male patients were more affected and the most prevalent age group was 11-30 years.
2. Patients living in the mesoregion agreste paraibano benefited most of the healthcare service provided.
3. The most frequent reasons for going to the service were motorbike accident, physical aggressions and falls.
4. Facial fracture surgery prevail in the service, in that, fractures of the orbitozygomatic complex and mandible were the most prevalent; they were reduced by open surgery.
